# An extracted tetraploid wheat harbouring the BBAA component of common wheat shows anomalous shikimate and sucrose metabolism

**DOI:** 10.1186/s12870-019-1796-9

**Published:** 2019-05-07

**Authors:** Ruili Lv, Lei Han, Binbin Xiao, Chaoxia Xiao, Zongze Yang, Hao Wang, Huan Wang, Bao Liu, Chunwu Yang

**Affiliations:** Key laboratory of Molecular Epigenetics of Ministry of Education (MOE), Northeast Normal University, Changchun, 130024 China

**Keywords:** Polyploidy, Extracted tetraploid wheat, Subgenome, Metabolomics and proteomics, Gene expression, Phenotype abnormality

## Abstract

**Background:**

The BBAA subgenomes of hexaploid common wheat are structurally intact, which makes it possible to extract the BBAA subgenomes to constitute a novel plant type, namely, extracted tetraploid wheat (ETW). ETW displays multiple abnormal phenotypes such as massively reduced biomass and abnormal spike development, compared to extant tetraploid wheat with a BBAA genome. The genetic, biochemical and physiological basis underlying the phenotypic abnormality of ETW remains unknown.

**Results:**

To explore the biochemical basis of these phenotypic abnormalities, we analysed the metabolomic and proteomic profiles and quantified 46 physiological traits of ETW in comparison with its common wheat donor (genome BBAADD), and a *durum* tetraploid wheat cultivar (genome BBAA). Among these three types of wheat, ETW showed a saliently different pattern of nutrient accumulation and seed quality, markedly lower concentrations of many metabolites involved in carbohydrate metabolism, and higher concentrations of many metabolites related to amino acids. Among the metabolites, changes in shikimate and sucrose were the most conspicuous. Higher levels of shikimate and lower levels of sucrose influence many metabolic processes including carbohydrate and amino acid metabolism, which may contribute to the phenotypic abnormalities. Gene expression assay showed downregulation of a shikimate degradation enzyme (5-enolpyruvylshikimate-3-phosphate synthase) coding gene and upregulation of several genes coding for the sucrose hydrolysis enzyme, which could explain the higher levels of shikimate and lower levels of sucrose, respectively.

**Conclusions:**

Our results suggest that significant and irreversible biochemical changes have occurred in the BBAA subgenomes of common wheat during the course of its co-evolution with the DD subgenome at the hexaploid level.

**Electronic supplementary material:**

The online version of this article (10.1186/s12870-019-1796-9) contains supplementary material, which is available to authorized users.

## Background

Polyploidy, or whole genome duplication (WGD), is a driving force in plant evolution, and all seed plants have experienced polyploidy at some point in their evolutionary histories [1–8]. In particular, many crops are neoallopolyploids that have undergone WGD concomitant with interspecific hybridization, i.e., allopolyploidy, in the recent past [9]. Ever since polyploid crops were domesticated, they have been bred and improved successfully. However, insufficient genetic diversity due to bottleneck effects inherent to recent allopolyploidization is a major hindrance to sustainable genetic improvement of allopolyploid crops. Thus, understanding the mechanisms of phenotypic alterations caused by polyploidy can help in further improvement of such crops [10].

Remarkably, common wheat (*Triticum aestivum* L.) as a very young allohexaploid species can adapt to wide-ranging geographic regions and diverse climatic and edaphic conditions across the globe [11]. Currently, 95% of the wheat cultivars are allohexaploid common wheat, and the remaining 5% are of the allotetraploid *durum* wheat (*T. turgidum* L.). It has been proposed that genome plasticity and coordinated dosage effects of the three constituent subgenomes are important contributing factors to the success of hexaploid common wheat [11]. Deeper understanding of the genetic, biochemical and physiological changes in the course of allohexaploid wheat evolution and domestication holds great promise for further wheat improvement.

Common wheat (*Triticum aestivum* L.) as formed by two allopolyploidization events. The first, which occurred 0.36–0.5 million years ago, resulted in the allotetraploid wheat, *Triticum turgidum* [11–13], and the second, which occurred about 8500–10,000 years ago, involved allohexaploidization between a domesticated allotetraploid wheat (closely related to the *durum* wheat) and an *Aegilops* species with the DD genome, i.e., *Aegilops tauschii*, resulted in the common wheat, *T. aestivum* [12]. Botanists have mimicked the second event by crossing and doubling the extant progenitor species of common wheat [14, 15]. In newly formed hexaploid wheat, interactions between BBAA genome and DD genome may immediately lead extensive homoeolog-specific changes in gene expression and DNA methylation and structural changes [14, 16–21]. Although alternative splicing and extensive silencing of homoeolog-specific expression were observed in some common hexaploid cultivars [22–24], A, B, and D subgenomes of the common wheat have remained largely intact in their structure [16, 25]. These characteristics of the hexaploid BBAA subgenomes pointed to its high integrity and potential functional independence, and make it possible to extract the BBAA component of the common wheat as an independent organism [16, 26]. This extracted allotetraploid wheat (ETW) provides an opportunity to assess the extent to which that BBAA component has been affected in terms of its phenotype, biochemical processes, and gene expression since the speciation of the common wheat.

Extracted allotetraploid wheat represents a ploidy-reversed (from hexaploid to tetraploid) form of tetraploid wheat [16]. The genome of ETW is identical to the BBAA components of its common wheat donor [16, 26] and its karyotype is highly stable [16]. Our earlier work [16] indicated that ETW shows significantly smaller biomass and anomalous gene expression than the natural tetraploid wheat cultivars; however, the biochemical basis of these phenotypic abnormalities has not been investigated. To elucidate the biochemical mechanisms underlying the phenotypic abnormalities of ETW, we assayed 46 physiological traits, > 500 metabolites and proteomic profiling of ETW, its common wheat donor, a tetraploid *durum* wheat cultivar, and a resynthesized allohexaploid wheat (genome BBAADD) obtained by crossing ETW as the maternal parent with an *Aegilops tauschii* line as the paternal parent [16]. This work attempts to identify the metabolic processes of the BBAA component of common wheat that have been modified during its evolutionary history as an allohexaploid, and which may provide useful clues for further wheat improvement.

## Results

### Growth and development

**Fig. 1:**
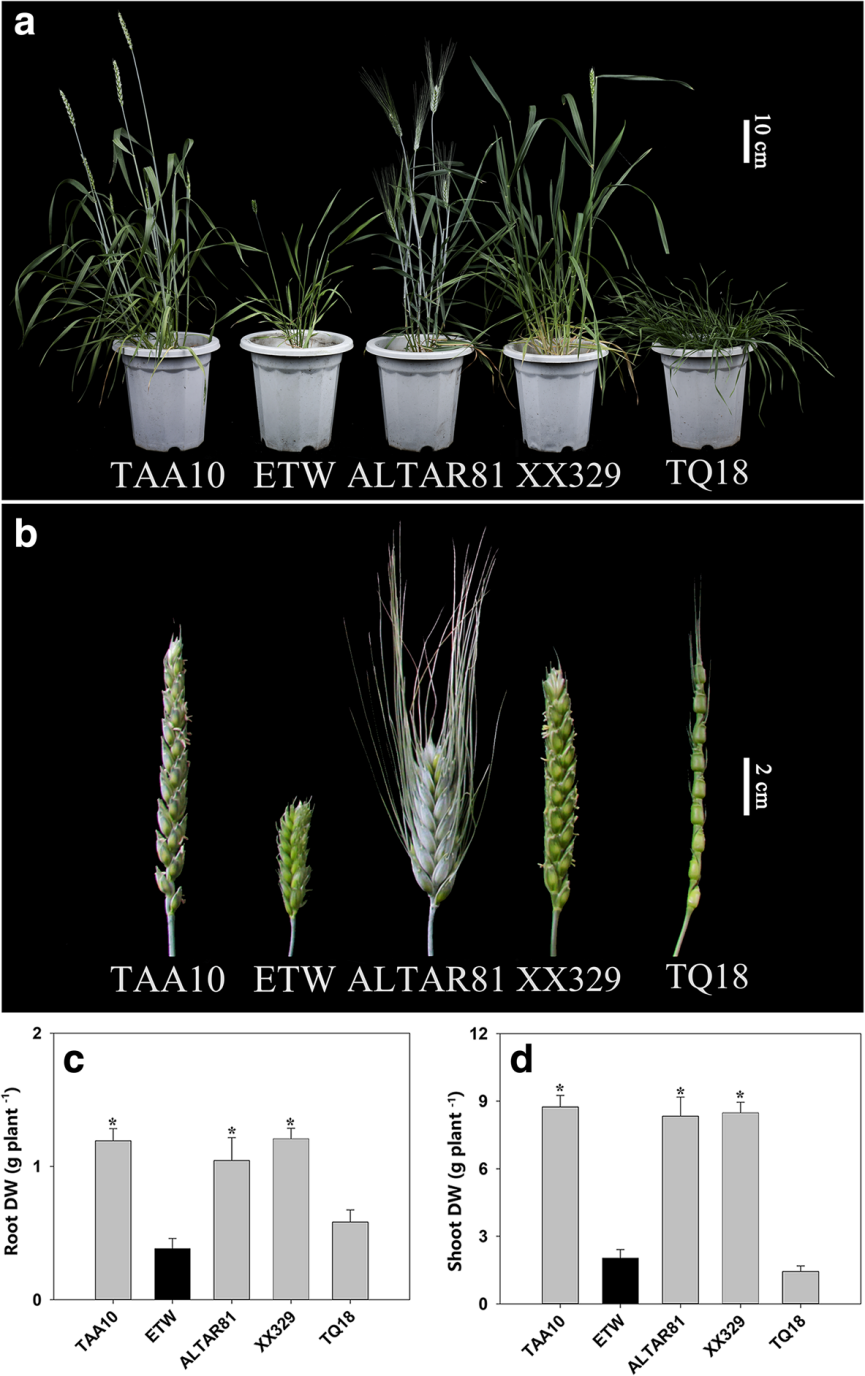
Phenotype of extracted tetraploid wheat (ETW), its common wheat donor (line TAA10, genome BBAADD), *Ae. tauschii* (line TQ18), an extant tetraploid wheat line (line ALTAR81, genome BBAA), and a resynthesized allohexaploid wheat (XX329, genome BBAADD) obtained by crossing ETW (maternal parent) and TQ18 (paternal parent). **a** Plants at flowering stage. **b** Spikes. **c** and **d** Dry weight. The dry weight values are means of 5–7 plants. Asterisks indicate significant differences (*t-*test, *P* < 0.05) between ETW and each wheat line

**Fig. 2:**
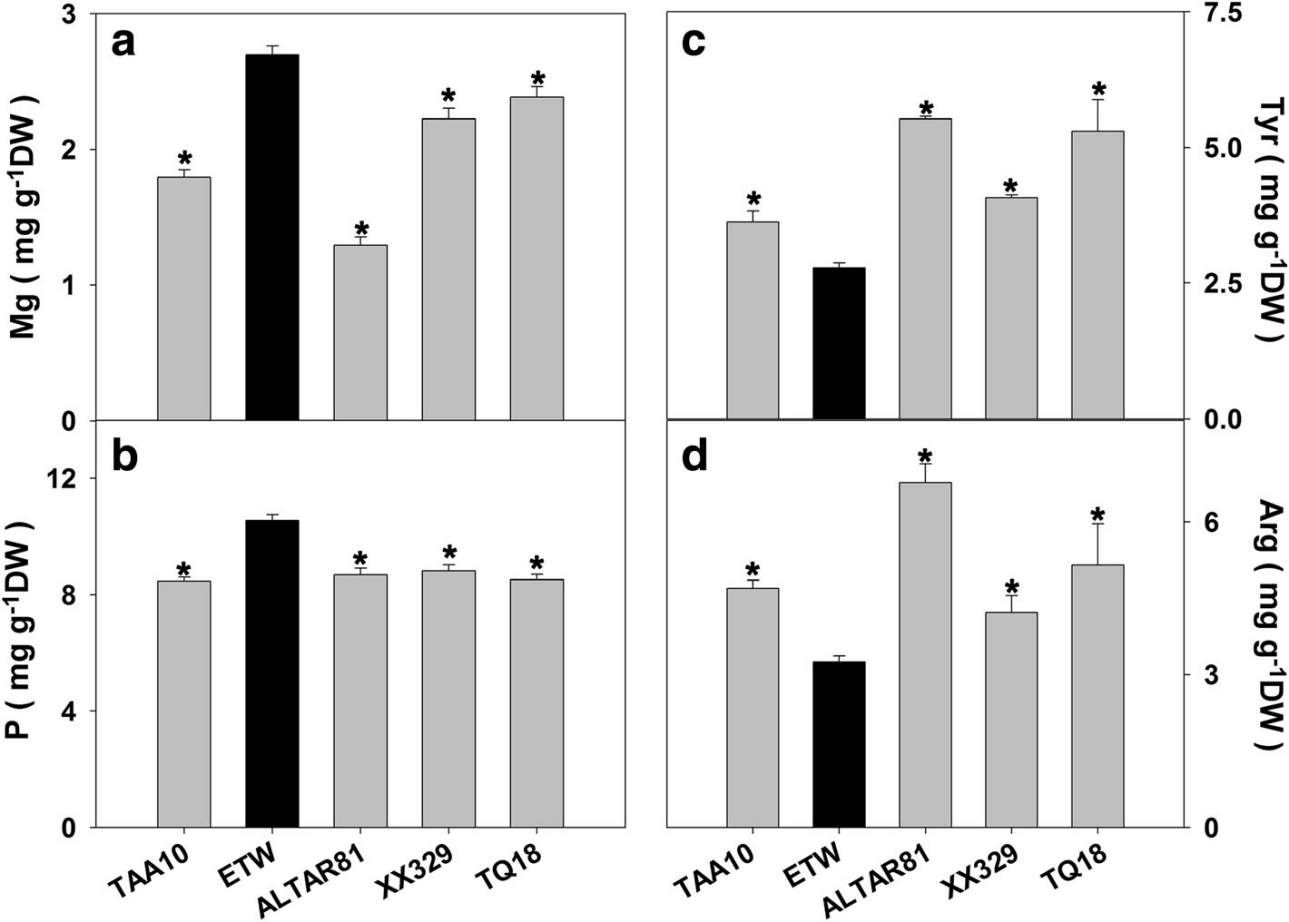
Contents of Mg (**a**), P (**b**), tyrosine (**c**), and arginine (**d**) in seeds of extracted tetraploid wheat (ETW), its common wheat donor (line TAA10, genome BBAADD), *Ae. tauschii* (line TQ18), an extant tetraploid wheat line (line ALTAR81, genome BBAA), and a resynthesized allohexaploid wheat (XX329, genome BBAADD) obtained by crossing ETW (maternal parent) and TQ18 (paternal parent). Asterisks indicate significant differences (*t-*test, *P* < 0.05) between ETW and each wheat line. Each genotype and each tissue had four biological replicates, and each biological replicate comprised a pool of five plants

We observed many phenotypic differences between ETW and the natural tetraploid wheat (line ALTAR81, labelled as AL) (Fig. 1 and Additional file 1: Figure S1). For example, ETW produced smaller leaves and its growth was slower (Fig. 1). However, all photosynthetic parameters and activities of three nitrogen metabolism enzymes (nitrate reductase (NR), glutamine synthetase (GS), glycolate oxidase (GO)) of ETW were similar to those of AL (Additional file 3: Table S1). We measured nutrient accumulation in shoots, roots and seeds and found that ETW and AL differed significantly in their contents of Ca, P, and K (Additional file 3: Table S1): ETW had higher concentration of Ca in shoots, higher concentrations of K and P in roots (Additional file 3: Table S1), and higher concentrations of P and Mg but lower concentrations of tyrosine and arginine in seeds (Fig. 2). We also observed defective development of spikes and seeds in ETW (Fig. 1 and Additional file 1: Figure S1). Interestingly, most phenotypic abnormalities of ETW can be restored by adding the DD genome back in the resynthesized hexaploid wheat XX329 obtained by crossing and doubling ETW and an *Aegilops tauschii* line (genome DD). For these defective phenotypes in ETW, XX329 showed similar traits to TAA10 and AL (Fig. 1 and Additional file 3: Table S1).

### Metabolomic profiles

**Fig. 3:**
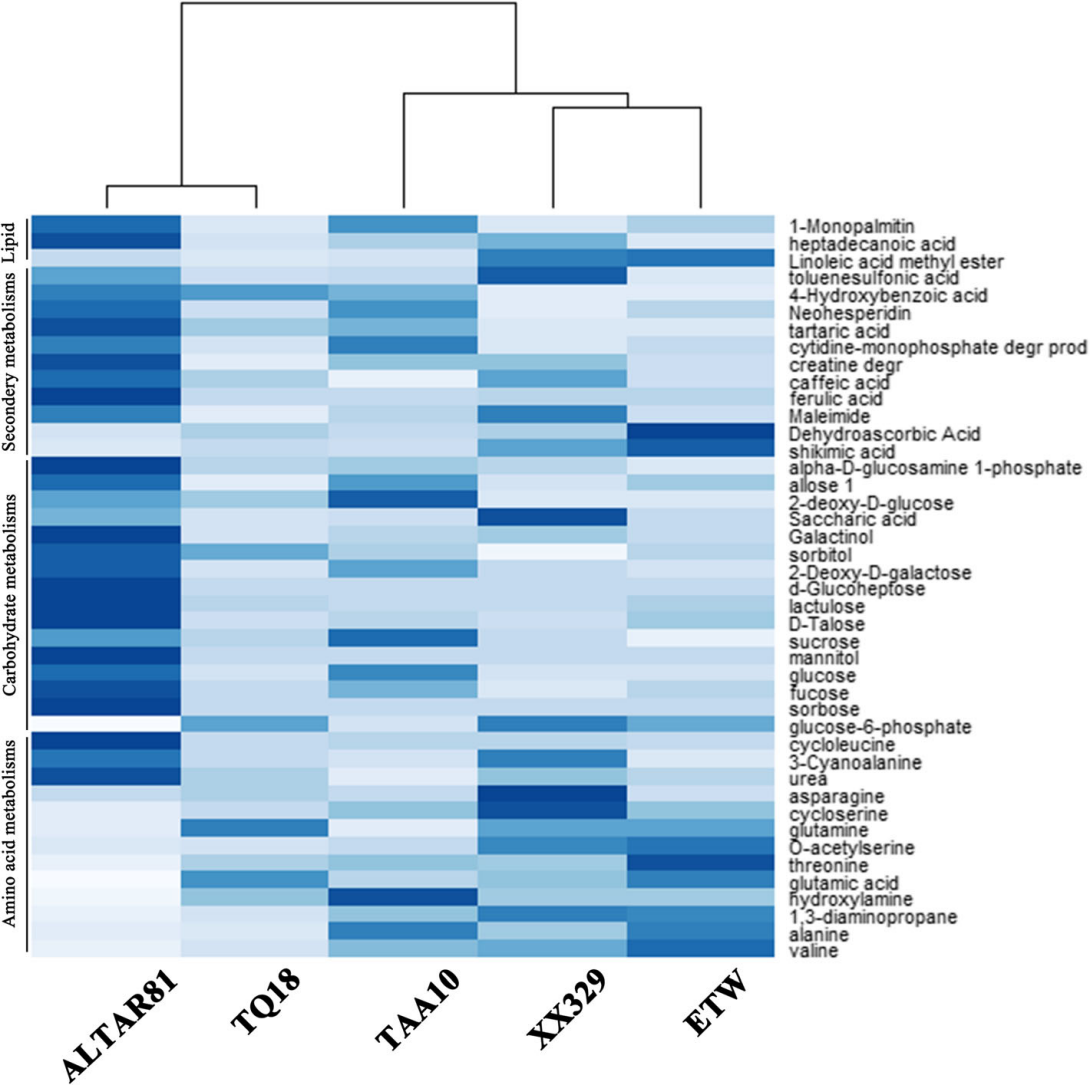
Heat map of metabolites of extracted tetraploid wheat (ETW), its common wheat donor (line TAA10, genome BBAADD), *Ae. tauschii* (line TQ18), an extant tetraploid wheat line (line ALTAR81, genome BBAA), and a resynthesized allohexaploid wheat (XX329, genome BBAADD) obtained by crossing ETW (maternal parent) and TQ18 (paternal parent). Only metabolites with twofold difference between ETW and AL are shown. For a given metabolite (row), the darker the shade, the greater the changes in concentration of the metabolites among the wheat lines. Each genotype had five biological replicates, and each biological replicate comprised a pool of 15 plants

**Fig. 4:**
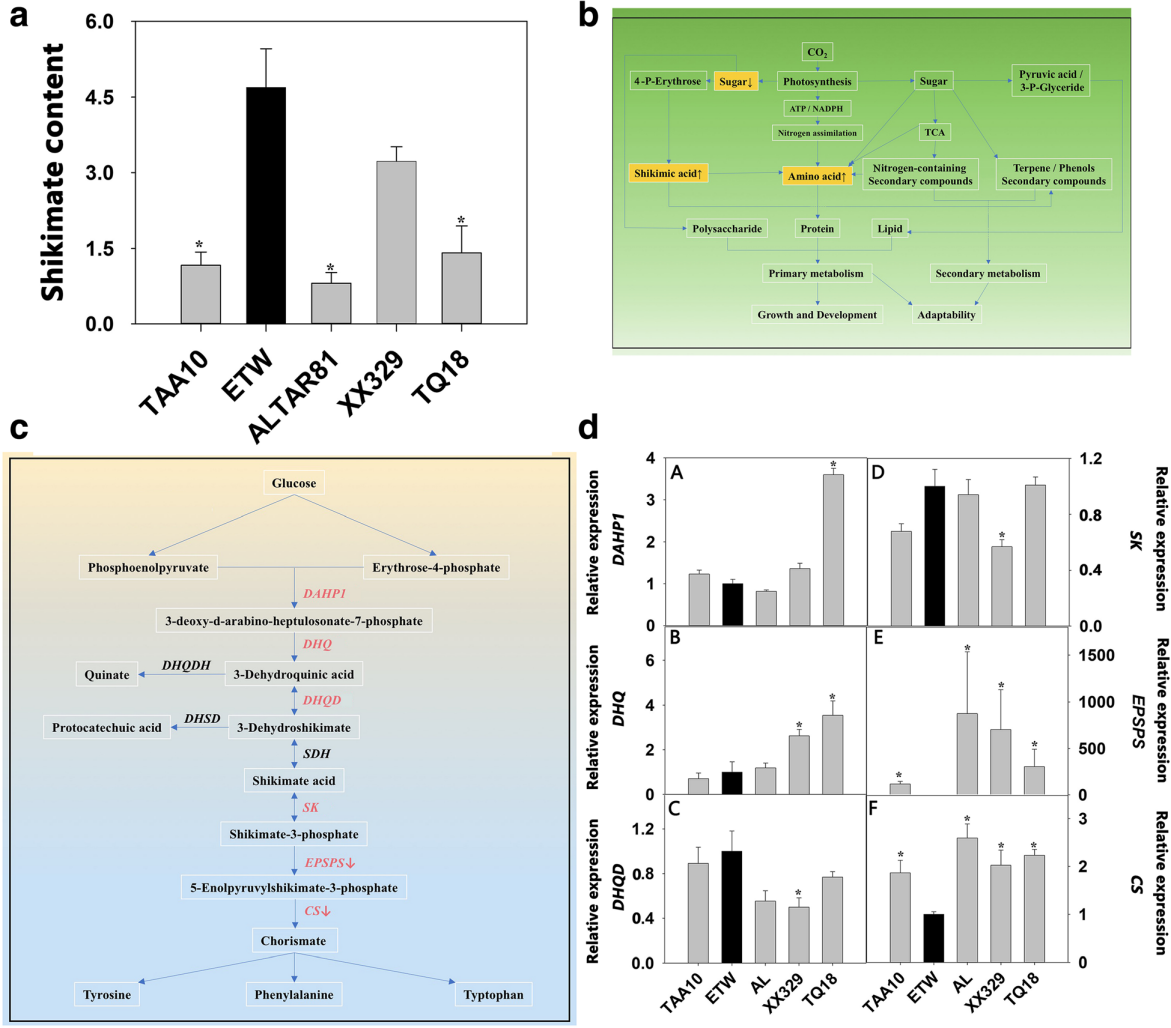
Differences of shikimate metabolism among extracted tetraploid wheat (ETW), its common wheat donor (line TAA10, genome BBAADD), *Ae. tauschii* (line TQ18), an extant tetraploid wheat line (line ALTAR81, genome BBAA), and a resynthesized allohexaploid wheat (XX329, genome BBAADD) obtained by crossing ETW (maternal parent) and TQ18 (paternal parent). **a** shikimate content. **b** Changes in metabolic network of shikimate in ETW. **c** Shikimate metabolism pathway. **d** Gene expression of shikimate metabolism pathway. The expressional values are means of four biological replicates. Asterisks indicate significant differences (*t-*test, *P* < 0.05) between ETW and each wheat line. *DAHP1*: 3-deoxy-7-phosphoheptulonate synthase 1, *DHQ*: 3-de-hydroquinic acid synthase, *DHQD*: 3-dehydroquinic acid dehydratase, *SK*: Shikimate kinase, *EPSPS*: 5-enolpyruvylshikimate-3-phosphate synthase, *CS*: Chorismate synthase

**Fig. 5:**
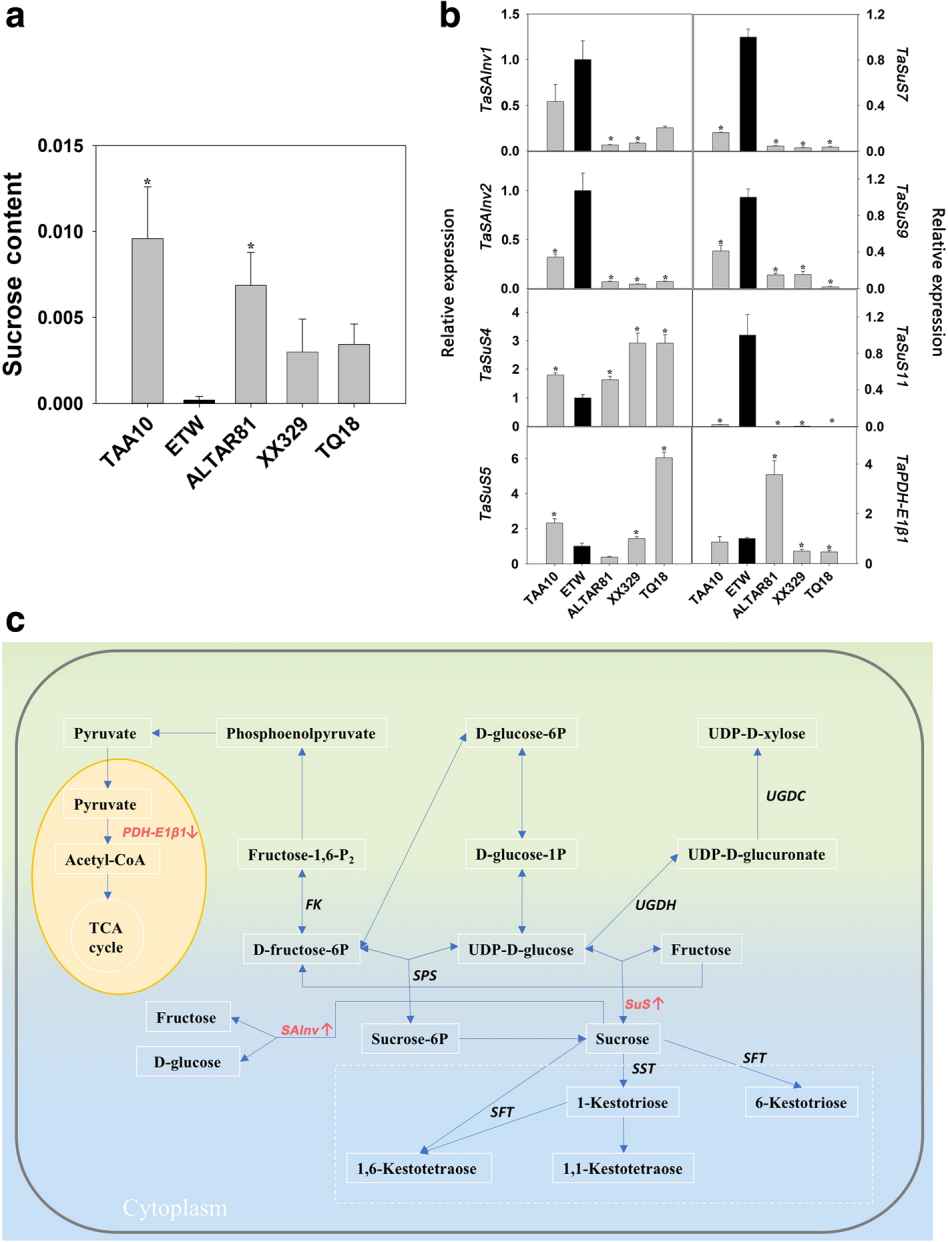
Differences in sucrose metabolism among extracted tetraploid wheat (ETW), its common wheat donor (line TAA10, genome BBAADD), *Ae. tauschii* (line TQ18), an extant tetraploid wheat line (line ALTAR81, genome BBAA), and a resynthesized allohexaploid wheat (XX329, genome BBAADD) obtained by crossing ETW (maternal parent) and TQ18 (paternal parent). **a** Sucrose content. **b** Expression of genes involved in sugar metabolism. The expressional values are means of four biological replicates. **c** Changes in sucrose metabolism network of ETW. Asterisks indicate significant differences (*t-*test, *P* < 0.05) between ETW and each wheat line. *SST*: Suc:suc fructosyltransferase, *SFT*: Suc:fructan fructosyltransferase, *PDH*: Pyruvate dehydrogenase, *UGDH*: UDP-Glc dehydrogenase, *UGDC*: UDP-glucuronate decarboxylase, *FK*: Fructokinase; *SPS*: Sucrose-P-synthase, *SuS*: Sucrose synthase, *SAInv*: Soluble acid invertase

To explore the biochemical basis of defects related to the growth and development in ETW, we compared the metabolomic profiles of ETW, AL, XX329, TAA10 and TQ18. We found that ETW is distinctly different from the rest four wheat genotypes in many metabolic pathways (Fig. 3 and Additional file 4: Table S2). We were particularly interested in metabolites that showed more than twofold differences between ETW and AL. Figure 3 and Additional file 4: Table S2 showed that contents of many metabolites involved in the metabolism of carbohydrates such as sucrose and glucose were much lower and those of several amino acid-related metabolites (glutamic acid, threonine, and O-acetylserine) were much higher in ETW (Additional file 4: Table S2). Of these metabolites, shikimate and sucrose showed particularly dramatic changes (Figs. 4 and 5). Shikimate content was much higher and that of sucrose was much lower in ETW than in all other four wheat genotypes (Figs. 4 and 5). We also observed some notable metabolic traits in XX329 (Additional file 4: Table S2). For example, the contents of threonine and 3-cyanoalanine were much higher or lower in ETW than in AL, TQ18 and TAA10, whereas in XX329 their contents had been restored to normal levels, close to those in AL and TAA10. However, ETW and AL differed in the levels of several metabolites, such as mannitol, glucose, sorbose, and tartaric acid, whereas ETW and XX329 did not, suggesting that the incorporated DD genome had not fully repaired the defects of ETW as far as these metabolites were concerned. In addition, XX329 differed from all the other wheat genotypes in some metabolites such as asparagine, cycloserine, and D-Talose, which indicates that the re-adding the DD genome to the BBAA genomes of ETW has resulted in the production of much more metabolites of certain types while much less of others.

### Gene expression and proteomics

The expression of six shikimate metabolism genes and 27 carbohydrate metabolism genes was analysed (Additional file 5: Table S3). The shikimate metabolism genes included *DAHP1*, *DHQ*, *DHQD*, *SK*, *EPSPS*, and *CS*, and the carbohydrate metabolism genes included two *SST* genes, two *SFT* genes, two *PDH* genes, one *UGDH* gene, one UGDC gene, three *CesA* genes, two *FK4* genes, six *SPS* genes, six *SuS* genes, and two *SAInv* genes. The breakdown on shikimate was catalysed by 5-enolpyruvylshikimate-3-phosphate synthase (EPSPS) (Fig. 4c); the gene encoding this enzyme was silenced in ETW but highly expressed in all other four wheat genotypes (Fig. 4d) although the other five genes (*DAHP1*, *DHQ*, *DHQD*, *SK*, and *CS*) involved in shikimate metabolism were expressed normally in ETW (Fig. 4d and Additional file 5: Table S3). Additionally, we found that the two gene families that catalyse sucrose hydrolysis, namely sucrose synthase (*SuS*) and soluble acid invertase (*SAInv*), were dramatically upregulated in ETW compared to AL and TAA10 (Fig. 5 and Additional file 5: Table S3). At least two *SAInv* genes (*TaSAInv1* and *TaSAInv2*) and three *SuS* genes (*TaSuS7*, *TaSuS9* and *TaSuS11*) were thus upregulated in ETW. For example, the expression level of *TaSuS11* in ETW was 7000 times of that in AL (Fig. 5 and Additional file 5: Table S3). Through proteomics analysis, we detected 1982 proteins, of which 219 were differentially expressed proteins (DEP) between AL and ETW (Table 1 and Additional file 6: Table S4); these were involved in DNA methylation, lipid metabolism, the antioxidase system, sugar metabolism and other metabolic processes (Table 1 and Additional file 2: Figure S2).

**Table 1:** Differentially expressed proteins (DEP) involved in DNA methylation, lipid metabolism, antioxidase system, and sucrose metabolism among the various wheat genotypes

Gene name	Fold change			Adjusted *P* value		
	AL/ETW	TAA10/ETW	XX329/ETW	AL/ETW	TAA10/ETW	XX329/ETW
DNA methylation						
Methyl-CpG binding domain	0.14	0.72	0.82	0.001	0.812	0.919
Methyl-CpG binding domain 1	0.18	0.96	0.64	0.013	1.000	0.620
Methyl-CpG binding domain 2	0.02	0.02	1.27	0.000	0.000	0.785
Lipid metabolism						
Lipoxygenase	0.02	0.02	5.15	0.000	0.000	0.000
Lipid transfer protein	0.14	0.20	0.48	0.001	0.000	0.181
Non-specific lipid-transfer	0.13	0.57	0.42	0.004	0.722	0.179
Non-specific lipid-transfer	0.15	0.09	0.30	0.044	0.000	0.054
Lipoxygenase	0.20	0.80	0.78	0.005	0.923	0.813
Lipoxygenase	0.26	1.74	3.47	0.029	0.125	0.000
Lipoxygenase	0.18	1.13	0.40	0.030	0.929	0.141
Antioxidase system						
Peroxidase	0.02	0.02	1.32	0.000	0.000	0.768
Ascorbate peroxidase	0.15	0.44	0.84	0.010	0.315	0.936
Peroxidase	0.09	0.28	0.47	0.000	0.007	0.231
Cu-Zn-SOD	0.25	1.49	2.19	0.026	0.361	0.013
Cu-Zn-SOD	0.18	0.97	1.42	0.001	0.989	0.499
Glutathione peroxidase	0.25	0.76	0.66	0.027	0.838	0.578
Glutathione S-transferase	50.00		50.00	0.000		0.000
Glutathione S-transferase	0.19	1.66	1.14	0.018	0.286	0.823
Glutathione S-transferase	0.24	0.92	0.81	0.018	0.989	0.869
Sugar metabolism						
Fructose-1,6-bisphosphatase	0.02	4.26	2.27	0.000	0.000	0.008
Fructose-1,6-bisphosphatase	0.02	4.30	2.57	0.000	0.000	0.005
Fructose-bisphosphate aldolase	0.02	5.70	4.25	0.000	0.017	0.006
Galactose-6-phosphate isomerase	0.02	0.02	0.91	0.000	0.000	0.992
Beta-fructofuranosidase	0.02	0.60	0.70	0.000	0.927	0.882
Glycosyltransferase	0.02	0.02	0.02	0.000	0.000	0.000
Glycosyltransferase	9.03	0.02	10.60	0.000	0.000	0.000
Beta-1,3-glucanase	0.03	0.02	0.06	0.000	0.000	0.000

The wheat genotypes used in this study include: extracted tetraploid wheat (ETW), its common wheat donor (line TAA10, genome BBAADD), a tetraploid wheat line (AL: ALTAR81, genome BBAA), and a resynthesized allohexaploid wheat (XX329, genome BBAADD) obtained by crossing ETW (maternal parent) and TQ18 (paternal parent). Only those proteins that showed at least a twofold difference were considered. Adjusted *P* values < 0.05. Only DEPs between ETW and AL are listed. Each genotype had four biological replicates, and each biological replicate comprised a pool of five plants

## Discussion

### The extracted tetraploid wheat showed defective growth and development

Whole genome duplication (WGD) can disrupt transcription and alter the expression level and pattern of homoeologs dramatically [1]. However, many homoeologs can retain their parental functions and expression patterns in neopolyploids [16]. The common wheat is a very young hexaploid and since its speciation, its BBAA component has been shaped by its co-resident DD component for nearly 10,000 years. The expression of BBAA homoeologs may have changed significantly, and some DNA fragments of the BBAA subgenome may even have been eliminated [14, 16–21]. The BBAA component of the common wheat can be extracted as a novel plant type, referred to as extracted tetraploid wheat, or ETW [16, 26]. Metabolic differences between ETW and natural tetraploid wheat can reveal the changes in the BBAA component that occurred during the evolutionary history of the common wheat. Our earlier studies identified many differentially expressed genes between ETW and natural tetraploid wheats (*Triticum turgidum*) [16, 18], studies that point to extensive transcriptional modifications after the speciation of the common wheat in its BBAA subgenomes. These previous findings led us to expect dramatic changes in phenotypes and metabolism processes in ETW as well. Indeed, ETW displayed multiple defective phenotypes such as massively reduced biomass and abnormal nutrient accumulation and spike development, compared to an extant tetraploid wheat (line ALTAR81, labelled as AL). However, most phenotypic abnormalities of ETW can be restored by adding the DD genome back in the resynthesized hexaploid wheat XX329 (Fig. 1 and Additional file 3: Table S1). Zhang et al. [16] also found that in XX329, most (> 96.1%) of the genes expressed differentially between ETW and natural tetraploid wheats showed additive expression, suggesting additional subgenome interactions after re-adding the DD subgenome is moderate. Although in theory the donor hexaploid wheat of ETW (TAA10) and XX329 have different DD subgenomes, the two hexaploid lines are highly similar in gene expression, indicating the intraspecific variations of *Ae. tauschii* are minimal [16]. These results also indicate that the extraction process per se has not resulted in anomalous phenotypes and metabolic processes of ETW, and therefore, the deteriorated phenotypes and altered metabolism are most likely due to evolved subgenome interactions in hexaploid wheat since its speciation.

### Metabolic profiling revealed anomalous shikimate and sucrose metabolism in ETW

Metabolic profiling displayed that ETW is distinctly different from other four genotypes in many pathways (Fig. 3 and Additional file 4: Table S2). ETW showed lower concentrations of many metabolites involved in carbohydrate metabolism, and higher concentrations of many metabolites related to amino acids. Of these metabolites, changes in shikimate and sucrose were particularly conspicuous (Figs. 4 and 5). Shikimate is a link between primary (basic) metabolism and secondary metabolism as well as between carbon metabolism and nitrogen metabolism (Fig. 4), and sucrose is central to carbon metabolism and photosynthesis and an important source of carbon for growth, development, and response to environment stress in wheat [27]. In ETW, increased shikimate content and decreased sucrose content may influence many metabolic processes including carbohydrate and amino acid metabolism, leading to abnormal phenotypes and impaired fitness (Figs. 4 and 5).

To gain some insights into the molecular basis of the anomalous metabolism of shikimate and sucrose in ETW, the expression of key genes involved in shikimate and carbohydrate metabolism was analysed (Additional file 5: Table S3). The results showed that shikimate breakdown gene (*EPSPS*) was silenced in ETW but highly expressed in all other four wheat genotypes (Fig. 4d and Additional file 5: Table S3). We propose that silencing of *EPSPS* gene facilitates the accumulation of shikimate in ETW (Fig. 4c). Additionally, we examined sucrose metabolism by the process described by Xue et al. [27], and found that the two gene families that catalyse sucrose hydrolysis (*SuS* and *SAInv*) were dramatically upregulated in ETW (Fig. 5 and Additional file 5: Table S3). High expression levels of sucrose hydrolysis enzyme genes may promote the degradation of sucrose in ETW, finally reduces the accumulation of sucrose in the leaves. Taken together, ETW shows abnormalities in two core metabolic processes that are part of shikimate and sucrose metabolism, abnormalities that, in turn, may affect many other related metabolic processes. Indeed, protein expression in many pathways in ETW showed extensive changes (Table 1 and Additional file 2: Figure S2), which may be attributable to the impaired metabolism of shikimate and sucrose or other related mechanisms. For instance, levels of three methyl-CpG binding proteins (core components involved in DNA methylation) were abnormal in ETW (Table 1), suggesting that the abnormal metabolic processes and defective phenotypes seen in ETW may be rooted to compromised DNA methylation and other epigenetic modifications, which warrant further studies.

## Conclusions

Compared with its hexaploid wheat donor and natural tetraploid wheat, ETW displayed many anomalous metabolic processes, suggesting that the metabolic processes of the BBAA component of common wheat have been modified during its evolutionary history. Among the metabolic processes, shikimate and sucrose metabolisms were the most conspicuous. The decreased sucrose level and increased shikimate level seen in ETW may profoundly affect its basic and secondary metabolisms, leading to abnormal phenotypes and impaired fitness. The data of gene expression provide a reasonable molecular explanation for the anomalous shikimate and sucrose levels of ETW, and revealed that the metabolic defects of ETW may be related to downregulation of a shikimate degradation enzyme gene and upregulation of several sucrose hydrolysis enzyme genes. We propose that, during evolutionary history of hexaploid wheat, expression pattern innovation of key genes may drive evolution of related core metabolic processes, and which may contribute to remarkable adaptability of hexaploid common wheat as globally the most important staple food crop.

## Methods

### Plant materials and growth conditions

The process of constructing of ETW is described in two publications [16, 26]. Briefly, ETW was constructed from BBAA components of a common wheat (*T. aestivum* L.) cultivar (“Canthach” referred to as TAA10 in the present paper) by crossing the common wheat with *durum* wheat (*T. turgidum*, genome BBAA), followed by repeated backcrossing with TAA10 as the recurrent parent. The genome of ETW is therefore virtually identical to the BBAA subgenome of its common wheat donor [16]. The resynthesized hexaploid wheat (XX329, BBAADD genome) was synthesized by crossing and doubling of ETW with an *Aegilops tauschii* (TQ18, DD genome) line developed by E. Kerber [26]. We used S6 generation plants for XX329 and ETW. A typical *durum* wheat line ALTAR81 (*T. turgidum*, BBAA genome) was used as a control. All five wheat lines were sown in pots filled with washed sand each containing one seedling. All seedlings were placed in a greenhouse. The growth conditions were maintained at 21–23 °C day and 14–17 °C night under16 h light at ~ 400 μmol m^− 2^ s^− 1^. The pots were watered daily with half-strength Hoagland nutrient solution. Initial seeds of ETW, TAA10, XX329 and TQ18 were kindly provided by Prof. Moshe Feldman of the Weizmann Institute of Science, Israel. Initial seeds of ALTAR81 were bought from International Maize and Wheat Improvement Center (CIMMYT).

### Sampling method and experiment design

We planted 100 pots for each genotype. At the tillering stage, we used 75 pots of plants of each genotype to conduct to proteomic analysis, biochemical measurements, metabonomic analysis, and Real-time PCR. We first collected the samples for metabonomic analysis. We took one mature leaf from each individual for each genotype, and each biological replicate was a pool of 15 leaves with five biological replicates. After sampling of metabonomic analysis, all the 75 pots of each genotype were divided into 3 sets (25 pots per set). The two sets (50 pots) were used for biochemical measurements and real time PCR, and another set (25 pots) for proteomic analysis. In addition, we used the remaining 25 pots of each genotype to measure seed quality, growth and photosynthesis. We arranged all genotypes based on randomized complete block design.

### Biochemical measurements

At the tillering stage, net photosynthetic rate (*P*_N_), stomatal conductance (*g*_s_), and transpiration rate (*E*) were measured by a portable open-flow gas exchange system (LI-6400, LI-COR, Lincoln, NE, USA). Leaves and roots of each genotype were collected at the tillering stage, and seeds of each genotype were collected at full ripe stage. Each genotype and each tissue had four biological replicates, and each biological replicate comprised a pool of 5 plants. Amino acids in seeds were separated and measured by an automated amino acid analyser [28]. Dried samples of leaves, roots, and grains were digested three times in 65% HNO_3_ at 120 °C, and their contents of Ca, Fe, Mg, P, and Na were measured using an inductively coupled plasma emission spectrometer. Enzymes in freshly matured leaves from each wheat line, all the leaves represented the same leaf position, were assayed using conventional methods [29–31]. The activity of nitrate reductase (NR), glutamine synthetase (GS), and glutamate dehydrogenase was measured according to methods described by Debouba et al. [29] and Surabhi et al. [30], and glycolate oxidase (GO) was assayed with the method described by Wu et al. [31].

### Metabonomic analysis

Mature leaves of 15 plants (one leaf per individual) at the tillering stage for each genotype were pooled as a biological replicate, and five such replicates were used in metabonomic analysis, which was conducted according to the method described by Guo et al. [32]. Briefly, leaf samples for each genotype were extracted in 0.4 mL of a mixture of methanol and chloroform (3:1, v/v). The extracted metabolites were derivatized with methoxylamine hydrochloride and N, O-bis (trimethylsilyl)-trifluoroacetamide (BSTFA) containing 1% trimethylchlorosilane (TMCS). Metabolite profiling was carried out using a GC-TOF/MS facility equipped with an Agilent 7890 gas chromatograph system and a Pegasus HT time-of-flight mass spectrometer (Chroma TOF Pegasus HT, Leco, Saint Joseph, MI, USA). Metabolic data were produced and analysed by Chroma TOF4.3X (a software package) and LECO-Fiehn Rtx5 database.

### Proteomic analysis

Leaves of five plants at the tillering stage for each genotype were pooled as a biological replicate, and four such replicates were used for each genotype. Total protein was extracted from fresh leaves in TCA-acetone solution (10% TCA in acetone), and the protein samples were stored in darkness at − 20 °C for 3 h. After centrifuging (13,000×*g*, 4 °C, and 30 min), the pellet was suspended in buffer A (8 M urea, 4% CHAPS, 30 mM HEPES, 2 mM Na_2_EDTA, 10 mM DTT, and 1 mM PMSF; pH 8.0–8.3). After centrifuging again as before, 35 μL of 1 M IAM and 15.8 μL 200 mM DTT were added to 0.3 mL of the supernatant. The protein samples were quantified by the Coomassie Brilliant Blue G250 method, digested in trypsin (trypsin: protein, 1:40) at 37 °C for 16 h, and the peptides were purified using Thermo Scientific Pierce C18 pipette tips (production ID 87784). Next, label-free proteomic analysis was conducted on an LC-MS/MS system (Q-Exactive, Thermo Scientific, Germany) according to the protocol stipulated by the manufacturer. Protein searching and label-free quantification were performed using Proteome Discoverer version 2.2 (Thermo Scientific, USA) against the common wheat reference genome database of the International Wheat Genome Sequencing Consortium (IWGSC) (iwgsc_refseq ver. 1.0). Differentially expressed proteins among the wheat genotypes were identified with ANOVA (background-based) method of Proteome Discoverer version 2.2, and the generated *p*-values were further adjusted by the Benjamini-Hochberg method.

### Real-time PCR

Total RNA from leaves at the tillering stage was isolated by TRIzol from Invitrogen, which is especially meant for plants, according to the manufacturer’s instructions. Each genotype had four biological replicates, and each biological replicate comprised a pool of five plants. The yield and quality of the RNA were checked by Nanodrop and agarose gel. The RNA was treated with DNaseI (Invitrogen), reverse-transcribed using SuperScriptTM RNase H-Reverse Transcriptase (Invitrogen), and then subjected to qRT-PCR analysis using gene specific primers (Additional file 7:Table S5). Real-time quantitative RT-PCR was performed using SYBR Green real-time PCR Master Mix and a StepOnePlus real-time PCR system. *Actin* and *RLI* were used as normalization control genes [33, 34]. The data of gene expression were analysed using the △△Ct method [35].

### Statistical analysis

The experimental design is randomized complete block design. All data were from 4 to 7 biological replicates. Statistical analysis for metabonomic and biochemical data was performed using SPSS version 13.0 (SPSS, Chicago, USA). The statistical significance was determined by the *t-*test at 0.05 level. Statistical analysis of proteomic data was performed using Proteome Discoverer version 2.2 based on ANOVA (background-based) method (adjusted *P* value < 0.05).

## Additional files

Additional file 1: **Figure S1.** Phenotype of spike of extracted tetraploid wheat (ETW) 10 days after flowering. (JPG 1177 kb)

Additional file 2: **Figure S2.** Frequency distribution of fold changes in protein abundance between extracted tetraploid wheat (ETW) and an extant tetraploid wheat line (AL, genome BBAA) and between ETW and a resynthesized allohexaploid wheat (XX329, genome BBAADD) obtained by crossing ETW (maternal parent) and TQ18 (paternal parent). (PDF 298 kb)

Additional file 3: **Table S1.** Differences in growth and biochemical traits among ETW, its common wheat donor (line TAA10, genome BBAADD), *Ae. tauschii* (line TQ18), an extant tetraploid wheat line (line ALTAR81, genome BBAA), and a resynthesized allohexaploid wheat (XX329, genome BBAADD) obtained by crossing ETW (maternal parent) and TQ18 (paternal parent). *E*: transpiration rate, GDH: glutamate dehydrogenase, *g*_s_: stomatal conductance, GO: glycolate oxidase, GS: glutamine synthetase, NR: nitrate reductase, *P*_N_: net photosynthetic rate. (XLSX 19 kb)

Additional file 4: **Table S2.** Contents and statistical information on metabolites differentially accumulated in ETW, its common wheat donor (line TAA10, genome BBAADD), *Ae. tauschii* (line TQ18), an extant tetraploid wheat line (line ALTAR81, genome BBAA), and a resynthesized allohexaploid wheat (XX329, genome BBAADD) obtained by crossing ETW (maternal parent) and TQ18 (paternal parent). Only those metabolites with at least twofold difference between ETW and AL are listed. (XLSX 19 kb)

Additional file 5: **Table S3.** Expression levels and statistical information on the genes involved in shikimate and sucrose metabolism of ETW, its common wheat donor (line TAA10, genome BBAADD), Ae. *tauschii* (line TQ18), an extant tetraploid wheat line (line ALTAR81, genome BBAA), and a resynthesized allohexaploid wheat (XX329, genome BBAADD) obtained by crossing ETW (maternal parent) and TQ18 (paternal parent). (XLSX 15 kb)

Additional file 6: **Table S4.** Abundance and statistical information on all differentially expressed proteins (DEP) between ETW and an extant tetraploid wheat line (line ALTAR81, genome BBAA). Only those proteins that showed at least a twofold difference were considered. Adjusted *P* value < 0.05. (XLSX 124 kb)

Additional file 7: **Table S5.** Primer sequences for real-time PCR analysis. (XLS 33 kb)

## Abbreviations

AL: ALTAR81
BSTFA: N, O-bis (trimethylsilyl)-trifluoroacetamide
*CesA*: Cellulose synthase
*CS*: Chorismate synthase
*DAHP1*: 3-deoxy-7-phosphoheptulonate synthase 1
DEP: Differentially expressed proteins
*DHQ*: 3-de-hydroquinic acid synthase
*DHQD*: 3-dehydroquinic acid dehydratase
*E*: Transpiration rate
*EPSPS*: 5-enolpyruvylshikimate-3-phosphate synthase
ETW: Extracted tetraploid wheat
*FK*: Fructokinase
GO: Glycolate oxidase
GS: Glutamine synthetase
*g*_s_: Stomatal conductance
IWGSC: International Wheat Genome Sequencing Consortium
NR: Nitrate reductase
*PDH*: Pyruvate dehydrogenase
*P*_N_: Net photosynthetic rate
*SAInv*: Soluble acid invertase
*SFT*: Suc:fructan fructosyltransferase
*SK*: Shikimate kinase
*SPS*: Sucrose-P-synthase
*SST*: Suc:suc fructosyltransferase
*SuS*: Sucrose synthase
TMCS: Trimethylchlorosilane
*UGDC*: UDP-glucuronate decarboxylase
*UGDH*: UDP-Glc dehydrogenase
WGD: Whole genome duplication

## References

[1] Chen ZJ (2007). Genetic and epigenetic mechanisms for gene expression and phenotypic variation in plant polyploids. Annu Rev Plant Biol.

[2] Jiao Y, Wickett NJ, Ayyampalayam S, Chanderbali AS, Landherr L, Ralph PE, Lynn P, Tomsho LP, Hu Y, Liang H (2011). Ancestral polyploidy in seed plants and angiosperms. Nature..

[3] Madlung A (2013). Polyploidy and its effect on evolutionary success: old questions revisited with new tools. Heredity..

[4] McCarthy EW, Chase MW, Knapp S, Litt A, Leitch AR, Le Comber SC (2016). Transgressive phenotypes and generalist pollination in the floral evolution of Nicotiana polyploids. Nat Plants.

[5] Fernández-Mazuecos M, Glover BJ (2017). The evo-devo of plant speciation. Nat Ecol Evol.

[6] MacKintosh C, Ferrier DEK (2017). Recent advances in understanding the roles of whole genome duplications in evolution. F1000 Res.

[7] Van DPY, Mizrachi E, Marchal K (2017). The evolutionary significance of polyploidy. Nat Rev Genet.

[8] Kellogg EA (2016). Has the connection between polyploidy and diversification actually been tested?. Curr Opin Plant Biol.

[9] Udall JA, Wendel JF (2006). Polyploidy and crop improvement. Crop Sci.

[10] Bharadwaj DN, Bahadur B, Venkat Rajam M, Sahijram L, Krishnamurthy K (2015). Polyploidy in crop improvement and evolution. Plant biology and biotechnology.

[11] Dubcovsky J, Dvorak J (2007). Genome plasticity a key factor in the success of polyploid wheat under domestication. Science..

[12] Feldman M, Lupton FGH, Miller TE, Smartt J, Simmonds N (1995). Wheats. Evolution of crop plants.

[13] Matsuoka Y (2011). Evolution of polyploidy Triticum wheats under cultivation: the role of domestication, natural hybridization and allopolyploid speciation in their diversification. Plant Cell Physiol.

[14] Feldman M, Levy AA (2012). Genome evolution due to allopolyploidization in wheat. Genetics..

[15] Li A, Liu D, Wu J, Zhao X, Hao M, Geng S, Yan J, Jiang X, Zhang L, Wu J (2014). mRNA and small RNA transcriptomes reveal insights into dynamic homoeolog regulation of allopolyploid heterosis in nascent hexaploid wheat. Plant Cell.

[16] Zhang H, Zhu B, Qi B, Gou X, Dong Y, Xu C, Zhang B, Huang W, Liu C, Wang X (2014). Evolution of the BBAA component of bread wheat during its history at the allohexaploid level. Plant Cell.

[17] Li AL, Geng SF, Zhang LQ, Liu DC, Mao L (2015). Making the bread: insights from newly synthesized allohexaploid wheat. Mol Plant.

[18] Wang X, Zhang H, Li Y, Zhang Z, Li L, Liu B (2016). Transcriptome asymmetry in synthetic and natural allotetraploid wheats, revealed by RNA-sequencing. New Phytol.

[19] Jung Y, Kawaura K, Mishina K, Sakuma S, Kishii M, Ogihara Y (2014). Changes in genome-wide gene expression during allopolyploidization and genome stabilization in hexaploid wheat. Genes Genet Syst.

[20] Leach LJ, Belfield EJ, Jiang C, Brown C, Mithani A, Harberd NP (2014). Patterns of homoeologous gene expression shown by RNA sequencing in hexaploid bread wheat. BMC Genomics.

[21] Qi B, Huang W, Zhu B, Zhong X, Guo J, Zhao N, Xu C, Zhang H, Pang J, Han F (2012). Global transgenerational gene expression dynamics in two newly synthesized allohexaploid wheat (*Triticum aestivum*) lines. BMC Biol.

[22] Bottley A, Xia GM, Koebner RMD (2006). Homoeologous gene silencing in hexaploid wheat. Plant J.

[23] Akhunova AR, Matniyazov RT, Liang H, Akhunov ED (2010). Homoeolog-specific transcriptional bias in allopolyploid wheat. BMC Genomics.

[24] Akhunov ED, Sehgal S, Liang H, Wang S, Akhunova AR, Kaur G, Li W, Forrest KL, See D, Simková H (2013). Comparative analysis of syntenic genes in grass genomes reveals accelerated rates of gene structure and coding sequence evolution in polyploid wheat. Plant Physiol.

[25] Jiang J, Gill BS (1994). Different species-specific chromosome translocations in *Triticum timopheevii* and *T. turgidum* support the diphyletic origin of polyploid wheats. Chromosom Res.

[26] Kerber ER (1964). Wheat: reconstitution of the tetraploid component (AABB) of hexaploids. Science..

[27] Xue GP, McIntyre CL, Jenkins CLD, Glassop D, Van Herwaarden AF, Shorter R (2008). Molecular dissection of variation in carbohydrate metabolism related to water-soluble carbohydrate accumulation in stems of wheat. Plant Physiol.

[28] Dluzniewska P, Gessler A, Dietrich H, Schnitzler JP, Teuber M, Rennenberg H (2007). Nitrogen uptake and metabolism in Populus×canescens as affected by salinity. New Phytol.

[29] Debouba M, Gouia H, Suzuki A, Ghorbel MH (2006). NaCl stress effects on enzymes involved in nitrogen assimilation pathway in tomato “*Lycopersicon esculentum*” seedlings. J Plant Physiol.

[30] Surabhi GK, Reddya AM, Kumaria GJ, Sudhakara C (2008). Modulations in key enzymes of nitrogen metabolism in two high yielding genotypes of mulberry (*Morus alba* L.) with differential sensitivity to salt stress. Environ Exp Bot.

[31] Wu TG, Gu SH, Zhou HF, Wang GG, Cheng XR, Yu MK (2013). Photosynthetic and physiological responses of native and exotic tidal woody seedlings to simulated tidal immersion. Estuar Coast Shelf S.

[32] Guo R, Shi LX, Yang CW, Yan CR, Zhong XL, Liu Q, Xia X, Li HR (2016). Comparison of ionomic and metabolites response under alkali stress in old and young leaves of cotton (*Gossypium hirsutum* L.) seedlings. Front Plant Sci.

[33] Ravel C, Martre P, Romeuf I, Dardevet M, El-Malki R, Bordes J, Duchateau N, Brunel D, Balfourier F, Charmet G (2009). Nucleotide polymorphism in the wheat transcriptional activator Spa influences its pattern of expression and has pleiotropic effects on grain protein composition, dough viscoelasticity, and grain hardness. Plant Physiol.

[34] Giménez MJ, Pistón F, Atienza SG (2011). Identification of suitable reference genes for normalization of qPCR data in comparative transcriptomics analyses in the *Triticeae*. Planta..

[35] Livak KJ, Schmittgen TD (2001). Analysis of relative gene expression data using real-time quantitative PCR and the 2 (−Delta Delta C(T)) method. Methods..

