# Supplementary figures and images for: An extracted tetraploid wheat harbouring the BBAA component of common wheat shows anomalous shikimate and sucrose metabolism
Source: BMC Plant Biol. 2019 May 7;19:188. doi: 10.1186/s12870-019-1796-9 (PMC6505309; doi:10.1186/s12870-019-1796-9)

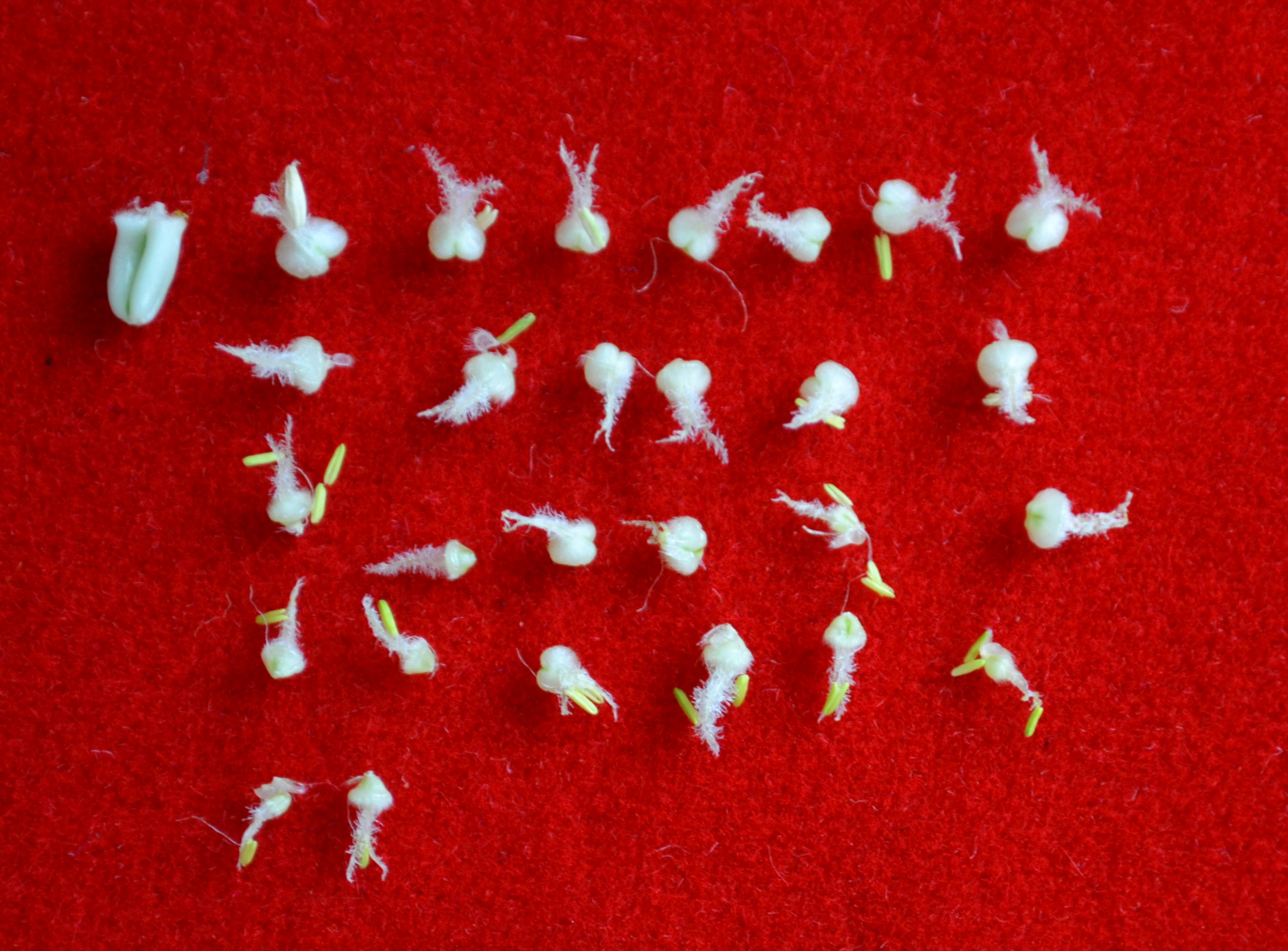

Supplement: Supplementary file 1 — Figure S1. Phenotype of spike of extracted tetraploid wheat (ETW) 10 days after flowering. (JPG 1177 kb) [file 12870_2019_1796_MOESM1_ESM.jpg]

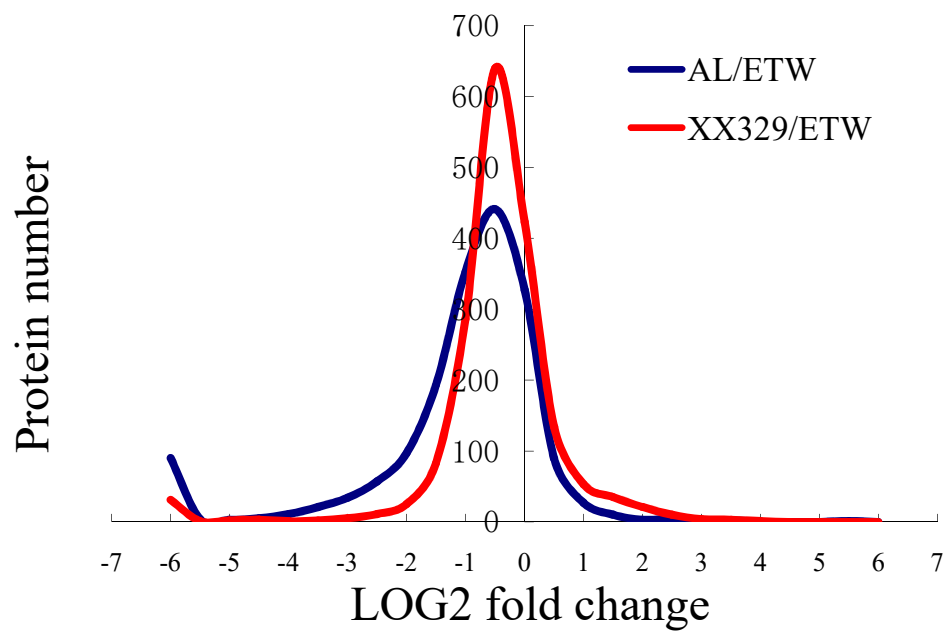

Supplement: Supplementary file 2 — Figure S2. Frequency distribution of fold changes in protein abundance between extracted tetraploid wheat (ETW) and an extant tetraploid wheat line (AL, genome BBAA) and between ETW and a resynthesized allohexaploid wheat (XX329, genome BBAADD) obtained by crossing ETW (maternal parent) and TQ18 (paternal parent). (PDF 298 kb) [file 12870_2019_1796_MOESM2_ESM.pdf]
